# Financing Externalisation: The Role That EU Funds Play in Shaping the Turkish Asylum and Migration Policies

**DOI:** 10.1080/15562948.2024.2407156

**Published:** 2025-01-03

**Authors:** Orçun Ulusoy

**Affiliations:** Amsterdam Center for Migration and Refugee Law, Vrije Universiteit Amsterdam, Amsterdam, The Netherlands

**Keywords:** Externalization, Financial aid 2016, EU-Turkey statement, migration management

## Abstract

The article investigates the impact of European Union (EU) financial instruments, particularly the Facility for Refugees in Turkey (FRIT), on Turkey’s migration and asylum policies. It critically examines how the EU-Turkey Statement of 2016 signaled a shift from Europeanisation to a more transactional relationship centered on externalization and migration containment. The paper argues that while these financial mechanisms provided crucial support during the refugee crisis, they also contributed to the selective Europeanisation and De-Europeanisation of Turkey’s policies, ultimately hindering the development of long-term structural migration policies.

## Introduction

The financial instruments of the European Union, specifically the Facility for Refugees in Turkey (FRIT), have been significant tools in shaping Turkey’s migration policies. Established as the financial instrument to deliver on the EU’s commitment under the EU-Turkey Statement and with the primary objective of managing the refugee crisis, the FRIT has been instrumental in providing financial aid to Turkey, thereby influencing its approach toward migration and asylum policies.

Since 2015, the Syrian Civil War has led to a significant influx of refugees into Turkey, making it host to the largest refugee population in the world. This sudden and massive influx posed significant challenges to Turkey’s existing migration and asylum framework, necessitating a comprehensive and effective response.

In this context, the FRIT emerged as a crucial mechanism after 2015, providing much-needed financial support to Turkey to manage the crisis. The funds from FRIT have been used in various sectors, such as education, health, infrastructure, and socio-economic support for refugees. This has helped not only address the immediate needs of the refugees but also their long-term integration into Turkish society.

However, the impact of FRIT funds extends beyond immediate humanitarian assistance. It has significantly influenced the way Turkey formulates and implements its migration policies. The availability of FRIT funds has slowed down the development of long-term, structural policies and led to permanent temporariness (Şanlıer, 2023, p. 45).

Moreover, the FRIT has also influenced Turkey’s cooperation with the European Union on migration issues. The EU-Turkey statement of 2016, which was hailed as a game-changer by Merkel, was aimed at ending irregular migration from Turkey to the EU and supporting Syrian refugees in Turkey (Dimitriadi et al., 2018). However, the statement did not provide migrants with decent and legal opportunities to move but rather changed the nature of cooperation between the EU and Turkey and legitimized migrants as a bargaining chip in international relations (Okyay & Zaragoza-Cristiani, 2016).

This article aims to delve deeper into the impact of FRIT funds on Turkish migration policies. It seeks to explore how these funds have shaped Turkey’s response to the refugee crisis, the changes that have been brought about in its migration and asylum policies, and the implications of these changes for the refugees and for Turkey. Through this exploration, the article hopes to provide a comprehensive understanding of the complex interplay between international aid, national policies, and the lived realities of refugees.

This article will start by providing an overview of the migration policies in Turkey and the EU, as well as their relations since the 2000s. It will then detail the EU financial instruments in Turkey, including the Facility for Refugees in Turkey (FRIT), which is a mechanism for managing funding to support refugees and host communities in Turkey. Finally, the article will discuss the impact of this assistance on the Turkish migration system, as well as on migrants and refugees in Turkey.

## Methodology

This article is based on the analysis of documents and data collected during the ASILE H2020 research project between 2021 and 2022. The ASILE project^1^ studies the interactions between emerging international protection systems and the United Nations Global Compact for Refugees (UN GCR), with a particular focus on the role of the European Union. Adopting an interdisciplinary perspective, it examines the characteristics of international and country-specific asylum governance instruments and arrangements and their compatibility with international and regional human rights and refugee laws.

Within the research project, the author was a member of a research team responsible for conducting the research on Turkey. Between June—October 2021, a total of 25 interviews were held with respondents working for governmental, international, and non-governmental organizations working on migration issues, and an in-depth literature review was conducted (Ovacik et al., 2022, p. 15).

Except for one, all interviews were conducted online due to the ongoing COVID-19 pandemic at the time of the research. Interviewees were selected from governmental, international and non-governmental organizations that have different degrees of involvement in the preparation and implementation of EU-Turkey Instruments on migration and asylum. Out of 25 interviews, nine respondents are from governmental organizations, eight work in international organizations, and eight are involved in non-governmental organizations.^2^

Official documents and press releases since the 2016 EU-Turkey Statement were the starting points for the document review. However, the most important and in-depth data was extracted from monitoring and financial audit reports of the (inter)governmental agencies involved in the financial aid. Finally, ever-growing academic literature focusing on the linkage between externalization and financial aid was also included in the literature review.

The ASILE research on Turkey collected and analyzed data focussing on the political, legal, and financial instruments and programmes through which the EU and Turkey have cooperated in the field of migration and asylum between 2015 and 2021. In this article, only financial instruments and their impact on the Turkish migration system will be discussed.

## Turkish Migration and Asylum policy history

In 1951, Turkey was one of the first nations to sign the Convention Relating to the Status of Refugees (hence, the 1951 Convention). Turkey not only actively participated in the drafting of the Convention (Weis, 1990) but also became a member of the Executive Committee of the High Commissioner’s Programme (ExCom), which was established in 1958 by the Economic and Social Council of the United Nations (ECOSOC) [Resolution 672 (XXV)]. The 1951 Convention was the first international agreement that defined the word “refugee” and outlined the rights and responsibilities of refugees and nations.

Turkey is one of four countries to retain the ‘Geographical Limitation’ clause of the 1951 Convention, despite having participated in the drafting and signing of the 1951 Convention and its 1967 Protocol (Baban et al., 2017, p. 1). According to this clause, Turkey declares that it would extend its legal duties solely to “European asylum-seekers” under the 1951 Convention. Non-Europeans are granted temporary protection, but neither long-term residency nor refugee status is permitted in Turkey. The United Nations High Commissioner for Refugees (UNHCR) was charged with processing the asylum petitions of non-Europeans in Turkey and coordinating their relocation to third countries.

Turkey’s reasons for maintaining the geographical restriction are multifaceted. The worry of a huge inflow threatening the economic and social-cultural structure of the south-east areas of Turkey is one of them, as is the “[Turkish officials’] fear Turkey becoming a dumping ground for irregular migrants apprehended in the European Union.” (Kirişci, 2002, p. 20). Nonetheless, “national security” is perhaps the most crucial cause. According to Kirişci (2002, p. 9), military and security circles in Turkey are adamantly opposed to lifting the territorial limits of the 1951 Convention.

The Turkish approach to national security may be understood in light of the nation-building process that began with the establishment of the Turkish Republic in 1923 (Cizre, 2003; Karaosmanoglu, 2000). Greece claimed independence from the Ottoman Empire in 1822; nevertheless, the First World War (1914–1918) and the Greco–Turkish War (1919–1922) established the borders and the relations of modern-day Greece and Turkey. The population exchange between Turkey and Greece that followed the Convention Concerning the Exchange of Greek and Turkish Populations, signed in Lausanne on January 30 1923, was one of the most defining events in the process of nation-building. The exchange entailed the deportation of Greeks from Anatolia to Greece and Greek Muslims to Turkey (Long, 2008). Clearly, the forced population exchange separated national security interests and human rights. Long (2008) underlines that important political elites viewed the exchange as a contribution to the “long-term stability of the Aegean through the nationalisation of political power structures and hence the exclusion of diversity” as a result of the “unmixing of peoples.” The ‘successful’ execution of the Lausanne Treaty allowed the Turkish government to conduct nation-building reforms without the fear of internal minorities or external actions connected to their protection (Oran, 2007). Due to the intimate relationship between migration and national security issues (İçduygu & Aksel, 2013), the control of migration was delegated to bureaucratic elites and security forces, such as police and coast guards. In the absence of asylum and migration legislation, their responses to the entry of migrants would not be governed by current norms, and thus, they would not be accountable (Wissink & Ulusoy, 2016).

Turkey maintained this “legislative gap” until the mid-1990s, when two mass influxes forced Turkey to draft its first piece of legislation on asylum.

### Transition period: 1990s

Beginning in the 1990s, Turkey had two significant refugee influxes, one from Iraq and the other from the Balkans, as a result of the Yugoslavian Civil War (Kirişci, 2004). However, the Turkish government, consistent with its policy since the founding of the Turkish Republic, has resisted offering any long-term protection to these refugees and only granted ad hoc, temporary solutions. On the other hand, these two mass influxes, but especially the presence of the Iraqi Kurds, forced Turkey to draft its first asylum-related legislation piece (Wissink & Ulusoy, 2016).

The 1994 Regulation on Asylum is the first Turkish legal regulation drafted particularly for asylum-related concerns. With this law, the government formalized a procedure they had begun applying to non-European asylum seekers in July 1994. This was a significant departure from the former practice in which the UNHCR office in Ankara was the primary authority for determining the refugee status of non-Europeans (Kirişci, 2001, pp. 78–81).

Until 1994, the Turkish asylum policy was governed by the terms of the Law on Settlement (Number: 2510) and the Passport Law (Number: 5682), while the procedure for determining the refugee status of non-European asylum seekers was delegated to the UNHCR office in Ankara. With the implementation of the Asylum Regulation of 1994, a new system, known as the parallel procedure, began to be applied. Despite the absence of an express reference to UNHCR’s participation in the rule, the whole procedure was dependent on UNHCR’s refugee status determination (RSD) and resettlement efforts. In order to be eligible for resettlement in a third country, a non-European asylum seeker was required to register both with the Turkish government (police) and the UNHCR headquarters in Ankara. Prior to the implementation of the Law on Foreigners and International Protection (LFIP) in 2014, this notion of parallel process was the prevalent practice.

#### The Law on Foreigners and International Protection (LFIP)

Since the turn of the century, the Turkish migration and asylum system has been undergoing a process of Europeanization. Adoption of the National Program of Action for the Adoption of the EU Acquis (NPAA) in 2001 and the National Action Plan for Asylum and Migration (NAP) in 2005 established a road map for the Turkish government to align its asylum policy and practice with EU standards.

The adoption of the Law on Foreigners and International Protection (LFIP) in 2013 was one of the most significant milestones of the Europeanisation of the Turkish asylum and migration system. This new law, in line with EU regulations, protected essential rights for asylum seekers and refugees in Turkey and cleared the way for the establishment of the Directorate General for Migration Administration (DGMM), a civilian organization for the management of migration.

In fact, however, the approval of the new law and the establishment of a new civilian authority had minimal effect on migrant and refugee rights in Turkey. After the enactment of the new law, geographical restrictions continued to be actively implemented. Non-European asylum seekers and refugees were again denied the chance of a long-term solution and placed in legal limbo since they were unable to get refugee status in Turkey and integrate into society due to the limitations.

DGMM, a newly constituted civilian organization, critically lacked capability and knowledge. In May 2015, the organization was formally created, and most of its staff had no prior expertise in migration and asylum concerns, while the majority of the remaining officials were from the Turkish Foreigners Police (Ulusoy, 2016). At the end of 2016, more than two years after the law and in the year that the EU-Turkey Statement was announced, supporting entities and processes, such as appeal committees and the country of origin information system, as well as secondary laws, were completely absent or were still in the process of being established (Ulusoy, 2016).

It is crucial to underline that the roadmaps, legislative drafting and adoption processes, and the establishment of the Directorate General of Migration Management (DGMM) were all directly supported by the European Union. This support was not just symbolic but manifested in the form of substantial financial aid and technical assistance.

The Twinning Projects,^3^ in particular, played a pivotal role in shaping the migration and asylum system in Turkey during this period. These projects were not just short-term interventions but strategic initiatives aiming at long-term development and reform. They were instrumental in several key areas, including the development of a national asylum and migration strategy and integrated border management.^4^ Indeed, the majority of these projects had a far-reaching impact that extended beyond their immediate implementation period. Notably, unlike the financial aid provided after 2016, these projects were mainly focused on institution building rather than ‘containment’.

### Post-Deal (2016 to 2023)

While the EU-Turkey Statement has been widely debated by the public since early 2016, discussions on a comprehensive readmission agreement between Turkey and the EU were initiated by the EU in November 2002 and were formally opened in May 2005.^5^ After four rounds of official discussions, the negotiations were nonetheless stopped in December 2006, when the EU decided to suspend the opening of several chapters with Turkey (Kirişci, 2014). In December 2009, parties returned to the negotiating table, and in June 2012, after three years of negotiations in Ankara and Brussels, a final document was developed and initiated.^6^ In December 2013, the final text of the agreement was signed in Ankara.^7^

However, the long-expected and negotiated readmission agreement did not work as planned. Just like the 2001 Greek-Turkish Readmission Protocol that aimed at the same readmission programme before the agreement, there were no significant readmission practices until the March 2016 EU-Turkey Statement. The failure in practice had multiple reasons, ranging from the political unwillingness to technical difficulties (İçduygu & Aksel, 2014, pp. 350–356).

Just three years before the EU-Turkey Readmission agreement, in 2010, Greece and Turkey reactivated the 2001 readmission treaty they had previously signed. In January 2011, the first group of 40 refugees was readmitted to Turkey, with Greece claiming that they were all Syrian nationals. However, the screening and identification procedure in Turkey following the group’s readmission revealed that only three of the forty readmitted migrants were Syrian nationals, while the remainder were from various nations, including a Turkish national.^8^ This was the initial and final attempt to restart the official readmission process between Greece and Turkey.

In 2016, at the height of the extraordinary migration from Middle Eastern and African nations to Europe, European officials, under pressure from the new arrivals, decided to renegotiate the Readmission Agreement with Turkey. After numerous rounds of intense discussions, the EU and Turkey reached an agreement on major practical concerns to minimize irregular migration to the EU on March 18, 2016.^9^ The suggested instruments roughly fall into two categories: a) provisions on an enhanced version of the EU-Turkey Readmission Agreement and b) incentive features to encourage Turkey to sign and execute the deal (Gkliati, 2017, pp. 87–88).

As incentive measures for Turkey, the EU’s allocation of substantial financing (up to 6 billion Euros) for refugees in Turkey, the acceleration of the visa liberalization plan, and the revitalization of EU membership negotiations were mentioned in the statement. The readmission agreement stipulates three operational steps as part of the 2016 EU-Turkey Agreement. First, Turkey is required to readmit any irregular migrants who crossed into Greece from Turkey. This requirement covers asylum applicants whose asylum petitions were pronounced inadmissible. The second is the relocation of Syrian refugees from Turkey to the European Union. EU is obligated to relocate the same number of Syrian refugees from Turkey as were repatriated from the Greek islands to Turkey, also known as the 1:1 scheme. After the irregular migration route between Turkey and Greece has been ‘brought under control’, the “Voluntary Humanitarian Admission Scheme” would be launched as the third phase.

Since the press release announcing the EU-Turkey Statement in Brussels, The Statement (or the Deal) has generally been seen as a ‘questionable externalization policy’ (Andersen et al., 2020) of the European Union. Moreover, while the politicians and drafters of this statement hail the agreement as a successful, game-changer instrument to control irregular migration, the reality in the field is far from being a success.

The statement is criticized for its negative impact on the human rights of migrants and refugees (Ulusoy & Battjes, 2017), the degrading consequences on the international migration law (Helme, 2021), instrumentalising the migrant lives in international politics and policy-making (Carrera et al., 2017), and its self-proclaimed success, which has been questioned by recent research (Tinni et al., 2023).

## EU Policies: From externalization to asylum as containment

As Turkey’s migration policies evolved after the 2000s, the European Union’s approach to migration also underwent significant changes. This section explores how the shift from Europeanisation to externalization within the EU’s migration policies, particularly following the EU-Turkey Statement of 2016, reflects broader dynamics of Europeanisation and De-Europeanisation.

Europeanisation refers to the process by which EU norms, policies, and practices are transferred to and adopted by member states and third countries, aligning their domestic policies with EU standards (Lavenex & Uçarer, 2004; Sedelmeier, 2011). Initially, the EU’s migration policy aimed to promote the adoption of these standards through instruments like readmission agreements, visa policies, and financial incentives, as part of a broader effort to ensure coherence and legitimacy within the Union.

The externalization of EU migration policies refers to the strategy of transferring the management of migration to non-EU countries, with the aim of preventing irregular migrants and persons in need of international protection from accessing the territory of the Member States (Moreno-Lax & Lemberg-Pedersen, 2019; Santos Vara & Pascual Matellán, 2021; van Munster & Sterkx, 2006). While the concept is new, as Crisp (2020) underlines, ‘…*the strategy is not particularly new* and has examples since the Nazi regime in the 1930s.’

Initially, the EU’s migration policy aimed to promote the adoption of common standards through instruments, as part of a broader effort to ensure coherence and legitimacy within the EU (Oliveira Martins & Strange, 2019). However, over time, the EU’s strategy shifted toward externalization—a policy approach where the management of migration is increasingly transferred to non-EU countries.

This shift is driven by the goal of preventing irregular migrants and asylum seekers from reaching EU territory, thereby securing the EU’s external borders. The externalization approach, while embedded within the broader Europeanisation framework, marks a departure from promoting EU norms toward prioritizing border control and containment strategies.

The EU-Turkey Statement of 2016 is a key example of this shift. While it can be seen as an extension of Europeanisation—encouraging Turkey to adopt practices aligned with EU migration control—it also exemplifies the transactional nature of EU-Turkey relations, where financial incentives are used to secure cooperation on migration management. This reflects a move away from the deeper normative influence of Europeanisation toward a more pragmatic approach focused on immediate security concerns.

Its also important to underline that Europeanisation and externalization are not necessarily opposites. They can go hand in hand as they are not mutually exclusive and often interact within the broader framework of EU migration governance. As we already experienced that the incentive for Turkey to adopt EU norms (Europeanisation) and externalization of migration policies have historically worked together. However, as mentioned above, the emphasis may have shifted in recent years. While the EU’s focus on externalization remains consistent, the importance placed on Turkey’s adoption of EU norms appears to have lessened.

De-Europeanisation is evident in this context as Turkey’s alignment with EU norms becomes increasingly selective. The transactional relationship fostered by the EU-Turkey Statement emphasizes containment over comprehensive integration and long-term structural alignment with EU standards. This selective Europeanisation undermines the EU’s normative power, reducing the impetus for Turkey to adopt EU-driven reforms in areas like human rights and long-term refugee protection.

The externalization of EU migration policies, while intended to ensure the functioning and legitimacy of the EU’s internal migration policy, has been criticized for being inconsistent, ineffective, and detrimental to human rights (Moreno-Lax & Lemberg-Pedersen, 2019; Spijkerboer, 2018; Tinni et al., 2023; Topak & Vives, 2018).

The main criticisms of this approach can be categorized into four themes:

*Lack of Coherence and Coordination:* The various policy instruments and actors involved in the external dimension of migration often result in conflicting objectives and divergent interests.

*Absence of Adequate Safeguards:* The transfer of migration control to third countries frequently lacks necessary safeguards, leading to human rights violations, particularly in countries with weak governance.

*Imbalance of Power:* The power dynamics between the EU and its partner countries often result in the imposition of the EU’s agenda without adequately considering the needs of third countries.

*Securitisation and Politicisation of Migration:* Migration is increasingly framed as a security threat, leading to policies that prioritize containment over the protection and integration of migrants and refugees.

While it was widely criticized, it also became one of the main aspects of the EU’s external migration policy framework such as (a) the Global Approach to Migration and Mobility (GAMM),^10^ which is the overarching framework for the EU’s dialogue and cooperation with third countries on migration and asylum issues, (b) the EU Agenda on Migration,^11^ which was adopted in 2015 in response to the migration crisis and aimed to provide a comprehensive and holistic approach to migration management, (c) the Migration Partnership Framework, which was launched in 2016 as a new way of engaging with key third countries of origin and transit of migrants and finally (d) the New Pact on Migration and Asylum,^12^ which was proposed in 2020 as a fresh start on migration in the EU, aiming to build on the previous initiatives and to address the gaps and shortcomings of the current system.

The ongoing evolution of the EU’s external migration policies intensified and “…arguably constitutes the most dynamic strand of EU migration policy, especially since the 2015/2016 asylum crisis in Europe.” (Niemann & Zaun, 2023, p. 2965). The EU-Turkey Statement can be seen as the ‘blueprint’ of this new path (Ineli-Ciger & Ulusoy, 2021), and the financial instruments accompanying the statement were the fundamental elements.

This approach, while effective in the short term, contributes to a process of de-Europeanisation by shifting the focus away from long-term normative goals and toward immediate security concerns.

This paper aims to contribute to the Europeanisation and De-Europeanisation literature by providing a critical analysis of the dynamic interplay between these processes in the context of EU-Turkey relations. It positions itself within the ongoing debate by illustrating how the EU’s migration policies have evolved from promoting Europeanisation to prioritizing externalization and containment.

The EU-Turkey relationship, particularly post-2016, exemplifies a form of selective Europeanisation that emphasizes security and containment over deeper structural alignment with EU norms. This shift toward a transactional relationship represents a form of de-Europeanisation (Kaya, 2021), where the influence of EU norms is diminished in favor of pragmatic, short-term solutions.

By examining the impact of EU financial instruments, such as the Facility for Refugees in Turkey (FRiT), the following sections highlight the complexities and contradictions inherent in the EU’s external migration policies.

## Financial instruments in Turkey

While collaboration between the EU and Turkey in the field of migration and asylum has a long history, the financial component of this cooperation has significantly increased and shifted since 2015. During the last decade, three major EU funding instruments, the Instrument for Pre-accession Assistance (IPA), the Facility for Refugees in Turkey (FRiT), and the EU Trust Fund in Response to the Syrian Crisis (Madad Fund) have provided substantial funding for asylum and migration-related activities in Turkey. While one of these tools, IPA, was created before 2015, the other two are direct EU responses to the continuing refugee crisis in the region (Ovacik et al., 2022).

The Instrument for Pre-accession Assistance (IPA) for Turkey was created by Council Regulation (EC) 1085/2006 on July 17 2006, and went into effect in January 2007.^13^ In an effort to match Turkish legislation and standards with those of the EU and to “increase the efficiency of the Community’s External Aid,” various current EU programmes and financial support mechanisms were replaced with a single legal framework and instrument. IPA I, which spanned the years 2007 to 2013, was planned to give financial aid *via* five channels (also known as the “components”): transition support and institution building, cross-border cooperation, regional development, human resource development, and rural development.^14^ Turkey was granted a total of 1,6 billion Euros. Between 2014 and 2020, the following instrument, IPA II, supplied Turkey with funds for capacity building in numerous areas in terms of EU acquis alignment and economic and social integration.^15^

With IPA II, the European Commission has adopted a sector-based framework as opposed to the previous IPA’s component-based structure. This funding sector has become the Facility for Refugees in Turkey (FRIT) following the October 2015 EU-Turkey Joint Action Plan. IPA II included funding for capacity building in the field of migration management, such as reception centers and the strengthening of the operational capacities of the Turkish Coast Guard.

The EU Regional Trust Fund in Response to the Syrian Crisis (Madad Fund), established in December 2014, is a humanitarian aid tool for Syrian refugees and their host nations.^16^ The Madad Fund is backed by twenty-one EU member states, together with Turkey and the United Kingdom, and focuses on financing large-scale programs in education, health, socio-economic support, and infrastructure. In January 2022, the total contracted Madad Fund projects amounted to 2.4 billion Euros. As an interesting situation, Turkey is both a donor and a recipient country of the Madad Fund, and 730 million Euros was allocated to initiatives in Turkey concentrating on education, livelihoods, and health.

## Facility for Refugees in Turkey

In October 2015, the EU and Turkey reached an agreement on a Joint Action Plan to “…intensify their support for Syrians under temporary protection and migratory management.”^17^ One month later, in November 2015, within this scope of cooperation, the European Commission announced the establishment of the Refugee Facility for Turkey (later renamed Facility for Refugees in Turkey - FRiT).

The Facility offers “a framework to coordinate the mobilisation of resources made available under both the EU budget and extra contributions from Member States that are integrated into the EU budget as external allocated revenues.” Therefore, the Facility is not a fund in and of itself but rather a coordinating vehicle for resource mobilization. The Facility encompasses humanitarian and development assistance, supported by several instruments—including the Humanitarian Aid Instrument (HUMA), the Instrument for Pre-Accession Assistance (IPA), and the Instrument for Stability and Peace (IcSP)—all of which were consolidated under the umbrella of FRIT after the EU Turkey Statement in 2016. As previously stated, FRiT has a total budget of €6 billion divided into two tranches, and financed initiatives are categorized as humanitarian and development. In addition, the Facility defined six priority areas: humanitarian aid, education, health, municipal infrastructure, socio-economic assistance, and migratory management. Humanitarian help includes providing monthly monetary assistance through the Emergency Social Safety Net (ESSN), as well as facilitating access to health care and education. On the other side, development projects strive to increase infrastructure and capacity through initiatives such as the building of schools and health facilities as well as vocational training and skill development.

The first tranche of €3 billion contracted between 2016 and 2017 included about €1.4 billion for humanitarian aid and approximately €1.6 billion for development-related measures. During the second tranche, the amount committed to development measures climbed substantially to €2 billion, while the amount allocated to humanitarian operations was reduced to €1 billion. Within the FRiT framework, 105 projects or activities were financed in two tranches. Sixty-two of these were classified as “Humanitarian” and 43 as “Development” (European Commission, 2023).

In addition, about half of the available cash under FRiT Tranche 1 was allocated to the initiatives of UN Agencies, such as WFP, UNHCR, and UNICEF. However, during the second tranche, the share of the UN Agencies drastically fell while the foreign organizations (such as the Red Cross), international financial institutions (World Bank) and Turkish authorities (Ministry of National Education) got the main portion of the financing.^18^

The “speed” of the FRiT’s implementation and the concentration of financed groups generated significant challenges concerning transparency, accountability, and the delivery of promised outcomes (Ovacik et al., 2022).

Interviewees emphasized the limited information available during the preparation stage of the FRiT. The majority expressed negative views regarding the overall transparency of the Instrument. For example, Interviewee 12, an international organization representative, asserted that there was a significant lack of transparency related to the design of the FRiT, which they attributed to a political decision driven by the pressure on the parties to act quickly. The focus of the FRiT was not transparent and was not readily available to their organization or the broader community in Turkey. Information was obtained on an ad-hoc basis, rather than in a systematic and structured manner. This lack of transparency was a source of frustration, as their feedback was not considered, and the only information accessible was what they could find on the website During the first tranche of the FRiT, the European Union financed two large initiatives under the priority area of migration management. With a combined budget of €80 million, these two initiatives were crucial to executing the EU-Turkey Statement from March 2016. The first of the two migration management projects is named “Support to the Implementation of the EU-Turkey Statement of March 18 2016”, executed by the Turkish Presidency of Migration Management (PMM) with a claimed budget of €60 million. The stated goal of the initiative was to “…assist Turkey in the management, receiving, and hosting of migrants, particularly irregular migrants found in Turkey and migrants repatriated from EU Member State territories to Turkey.”

The project comprised the building of a new temporary removal facility, the relocation of irregular migrants and Syrian refugees within Turkey, and the expansion of DGMM’s capability in the area of irregular migration. Due to the limited number of readmissions from Greece to Turkey under the EU Turkey Statement, just one “container” center with a capacity of 750 was constructed in Cankiri, Turkey. The funding was also intended to support “hosting and accommodating” irregular migrants in “appropriate conditions” by providing financial support for basic health care services, psycho-social services, translation services, food, hygiene, and other facilities essential to daily life and facility security. Lastly, financing was made available to support and expand GO-NET (the Turkish government’s database for migration management) with a database for irregular migration.

The second project was undertaken by IOM in collaboration with the Turkish Coast Guard (TCG) Command and is titled “Improving the Turkish Coast Guard’s Capability to Conduct Search and Rescue Operations.” With a budget of €20 million, the project involved the acquisition of six search and rescue vessels for the TCG, training for TCG personnel, and psychological assistance for TCG personnel to prevent burnout.

## The impact

The EU has been the main donor of humanitarian and development assistance to Turkey, especially in response to the Syrian refugee crisis and allocated €6 billion under the Facility for Refugees in Turkey (FRiT) to support the basic needs, education, health, socio-economic integration and protection of refugees and host communities as detailed above. These instruments have positively improved the living conditions, access to services and social cohesion of refugees and asylum seekers. Innovative and large-scale projects such as ESSN became a lifeline for many and lowered the pressure on local and national governments.

However, the EU’s financial mechanism also has significant challenges and negative impacts. These problems are directly connected to the way they were designed and implemented.

As underlined in a report by the European Court of Auditors (ECA), the contracting speed of the Facility compared to other EU Trust Funds is significant (European Court of Auditors, 2018, p. 49). The first tranche of the FRiT, €3 billion, was successfully distributed and made available to the implementers in less than two years. While this fast-forward approach can be justified as it was needed to distribute much-needed aid in the field as soon as possible, this speed also led to a centered decision-making process that excluded many actors and partners. This exclusion led to the limited involvement of civil society and local actors in the design, implementation, and monitoring of the EU-funded projects in Turkey, which reduced the interventions’ ownership, sustainability, and accountability.

Indeed, almost all interviewed civil society representatives underlined that their involvement in the designing period of the funding mechanism and guidelines was very limited. According to an international aid organization representative,^19^ in some cases, the implementing institution was pre-determined at the planning phase (Ovacik et al., 2022, p. 36). There is a need for a more participatory and inclusive approach to ensure the representation and empowerment of refugees and asylum seekers, as well as the local authorities and communities, in the decision-making and implementation processes of the EU-funded projects.

Another concern is the significant lack of internal monitoring and accountability mechanisms associated with these funds. While national authorities^20^ emphasize the existence of national accountability procedures to address potential violations, an international organization representative^21^ highlighted the absence of a specific system tied directly to EU funding. Both international organizations and civil society representatives^22^ have noted this gap, explaining that they rely on legal, formal, and informal follow-up through trust relationships at the local level. However, as an international civil society practitioner pointed out, because their work focuses on individual refugees rather than systemic issues, they lack direct communication channels with higher authorities to report potential human rights violations. Consequently, these violations or complaints are not systematically reported or addressed. However, the most critical impact of the EU’s financial instruments in Turkey has been on the evolution of the Turkish migration and asylum system. As discussed in the previous sections, before the 2015/2016 crisis, Turkey and the EU had some cooperation - albeit slowed down- on issues such as migration management, visa liberalization, readmission, and resettlement. On the other hand, after the 2016 deal, the nature of the relationship changed from a partnership to a transactional one, where the EU prioritized the containment and deterrence of irregular migration flows over the protection and integration of refugees and asylum seekers (Ovacik et al., 2022; Şanlıer, 2023; Tinni et al., 2023). An interviewee from an international organization described border management as a significant component of both IPA and FRIT, emphasizing that these instruments do not contribute to mobility. They further argued that, in pursuit of containment, the EU is employing a more subtle approach by outsourcing migration control, rather than simply erecting physical barriers.^23^ As another interviewee underlined, *“…although the [financial] instruments have containment purpose, it is not spoken of to avoid being politically incorrect”.*^24^

Naturally, this shift undermined Turkey’s incentives and prospects to adopt and implement the EU standards and norms on asylum and migration. It reduced the EU’s credibility as a human rights actor (van Heukelingen, 2021). As Kaya underlines, *“the EU–Turkey Statement is therefore rather an indication of Turkey’s de-Europeanization process”* (Kaya, 2021, p. 366).

## Conclusion

The European Union’s financial instruments, particularly the Facility for Refugees in Turkey (FRIT), have played a pivotal role in shaping Turkey’s migration and asylum policies in the wake of the Syrian refugee crisis. This paper has explored how these financial mechanisms, established as part of the EU-Turkey Statement of 2016, have not only provided essential humanitarian assistance but have also significantly influenced Turkey’s broader migration management strategies. The findings indicate that while FRIT and similar instruments have been instrumental in addressing the immediate needs of refugees, they have also contributed to complex and sometimes unintended consequences in Turkey’s migration governance.

One of the critical observations is the shift from a cooperative Europeanisation framework to a more transactional relationship between the EU and Turkey, focused on externalization and the containment of migration. This shift reflects a broader trend where the EU’s normative influence has been partially supplanted by pragmatic, short-term objectives. The reliance on financial incentives to secure Turkey’s cooperation has, in many ways, led to selective Europeanisation, where only certain aspects of EU norms are adopted, while others, particularly those related to human rights and long-term integration, are neglected.

Furthermore, the analysis reveals that the influx of EU funds has, paradoxically, slowed down the development of long-term structural migration policies in Turkey. The availability of substantial financial resources has encouraged a reliance on temporary measures and ad hoc solutions, rather than fostering comprehensive, sustainable policy reforms. This has led to a state of "permanent temporariness," where both refugees and the Turkish migration system are caught in a cycle of short-term fixes without meaningful progress toward lasting solutions.

The lack of robust internal monitoring and accountability mechanisms tied to EU funding has further complicated the situation. While national authorities assert the presence of accountability systems, the absence of specific mechanisms linked to EU funds has been a significant gap, as highlighted by both international and civil society organizations. This deficiency undermines the effectiveness of the financial support provided and raises concerns about the protection of human rights within the framework of these migration management strategies.

In summary, this paper underscores the dual-edged nature of the EU’s financial intervention in Turkey’s migration crisis. While it has undoubtedly provided much-needed support, it has also reinforced a policy environment that prioritizes containment over integration, and short-term gains over long-term sustainability. The findings suggest that for EU-Turkey cooperation on migration to be truly effective, it must move beyond the current transactional model toward a more holistic approach that balances immediate needs with the development of durable, rights-based migration policies. Only then can the EU and Turkey achieve a migration management system that is both effective and just, serving the interests of all stakeholders, with a primary focus on the people on the move.

## Notes

1 https://www.asileproject.eu/.

2 A further detailed methodology on data collection during the Covid-19 pandemic period, complete questionnaire as well as the (anonymized) list of interviewees can be found on https://www.asileproject.eu/d5-2_wp5-turkey-country-report-final/.

3 Review of Twinning in Turkey: Annex to the Main Report. European Neighbourhood Policy and Enlargement Negotiations. Last accessed on January 4, 2024; https://neighbourhood-enlargement.ec.europa.eu/system/files/2019-01/annexes_to_final_report_review_of_twinning_turkey.pdf.

4 One example of these project was the establishment of an Asylum and Country of Origin system, with the support of German, Swedish, Danish, and Dutch counterparts.

5 Proposal for a Council Decision of […] concerning the conclusion of the Agreement between the European Union and the Republic of Turkey on the readmission of persons residing without authorisation, COM/2012/0239 final-2012/0122 (NLE).

6 Proposal for a Council Decision of […] concerning the conclusion of the Agreement between the European Union and the Republic of Turkey on the readmission of persons residing without authorisation, COM/2012/0239 final-2012/0122 (NLE).

7 Agreement between the European Union and the Republic of Turkey on the readmission of persons residing without authorization OJ L 134, 7.5.2014, p. 3–27 http://data.europa.eu/eli/agree_internation/2014/252/oj.

8 Author’s own account. Author was monitoring the readmission procedures on behalf of a NGO.

9 European Council Press release, EU-Turkey statement, 18 March 2016; https://www.consilium.europa.eu/en/press/press-releases/2016/03/18/eu-turkey-statement/.

10 https://eur-lex.europa.eu/legal-content/EN/ALL/?uri=CELEX:52011DC0743.

11 https://eur-lex.europa.eu/legal-content/EN/TXT/?uri=CELEX%3A52015AE4319.

12 https://commission.europa.eu/strategy-and-policy/priorities-2019-2024/promoting-our-european-way-life/new-pact-migration-and-asylum_en.

13 Council Regulation (EC) No 1085/2006 of 17 July 2006 establishing an Instrument for Pre-Accession Assistance (IPA) https://eur-lex.europa.eu/legal-content/EN/TXT/HTML/?uri=CELEX:32006R1085&from=EN.

14 European Commission - Instrument for Pre-accession Assistance https://ec.europa.eu/neighbourhood-enlargement/enlargement-policy/overview-instrument-pre-accession-assistance_en.

15 European Commission, European Neighbourhood Policy and Enlargement Negotiations, Turkey - financial assistance under IPA II https://ec.europa.eu/neighbourhood-enlargement/enlargement-policy/overview-instrument-pre-accession-assistance/turkey-financial-assistance-under-ipa-ii_en.

16 EU Regional Trust Fund in Response to the Syrian crisis https://ec.europa.eu/trustfund-syria-region/index_en.

17 European Commission, Fact Sheet – EU-Turkey Joint Action Plan, Brussels, 15 October 2015, <http://europa.eu/rapid/press-release_MEMO-15- 5860_en.htm>.

18 List of projects funded by the Facility for Refugees in Turkey, as of 28 October 2023: https://ec.europa.eu/neighbourhood-enlargement/document/download/49484102-739e-407f-888d-fa7d2dc33879.

19 Interviewee TR14, (Ovacik et al., 2022, p. 36).

20 Interviewees TR4; TR7; TR9 ((National and Migration Practitioners).

21 Interviewee TR12, (Ovacik et al., 2022, p. 41).

22 Interviewees TR18; TR20; TR21; TR22 (Civil Society Practitioners).

23 Interviewee TR12, (Ovacik et al., 2022, p. 53).

24 Interviewee TR16, (Ovacik et al., 2022, p. 53).
